# Recurrent Ischemic Events in Adult Moyamoya Disease: A Case Report of Persistent Symptoms Despite Encephaloduroarteriosynangiosis (EDAS) Surgery

**DOI:** 10.7759/cureus.104906

**Published:** 2026-03-09

**Authors:** Huilan Tang, Shideh Doroudi, Bijal Sheth, Marian Ghaly, Christine Leroy

**Affiliations:** 1 Family Medicine, St. Joseph's Regional Medical Center, Paterson, USA; 2 Family Medicine, St. Joseph's Regional Medical Center, Clifton, USA

**Keywords:** cerebrovascular disorder, clinical case report, edas surgery, moyamoya disease, neurology and neurosurgery

## Abstract

Moyamoya disease (MMD) is a rare cerebrovascular disorder characterized by progressive stenosis of the intracranial arteries, which can lead to the development of fragile collateral vessels. The surgical revascularization, especially encephaloduroarteriosynangiosis (EDAS), is commonly employed among MMD patients. However, due to the slower development of collateral circulation in adult patients, the effectiveness of EDAS in adults has been questioned. Conversely, direct revascularization procedures, such as superficial temporal artery to middle cerebral artery (STA-MCA) bypass, have provided immediate restoration of blood flow and shown favorable outcomes among adult MMD patients. This report presents the case of a 39-year-old Hispanic female patient who was diagnosed with MMD and experienced multiple ischemic events despite EDAS. Subsequent imaging revealed progression of stenosis in both the anterior and posterior circulations. Given subsequent clinical deterioration, the patient opted for a right craniotomy for STA-MCA bypass. This surgical invention resulted in improved cerebral perfusion and stabilization of her neurological status. This case illustrates the complexity of managing adult-onset Moyamoya disease and suggests that indirect revascularization alone may not provide sufficient long-term hemodynamic stability in patients with advanced or progressive disease. These findings support consideration of direct or combined surgical approaches in carefully selected adult patients.

## Introduction

Moyamoya disease (MMD) is a rare progressive cerebrovascular disorder characterized by stenosis or occlusion of the intracranial arteries, leading to the development of fragile collateral vessels resembling the "puff of smoke" seen in angiography. Even though MMD majorly manifests among children, it is also often reported in adults in their mid-40s, with a female dominance [1].

Adult MMD patients typically present with ischemic symptoms, such as transient ischemic attacks and strokes, instead of hemorrhagic events [1]. Compared to pediatric MMD patients, the natural history of adult MMD has been less well studied. More recent studies have highlighted the progressive nature of MMD among adults. For example, a study conducted by Kuroda and Houkin (2008) has shown that approximately 20% of adult MMD patients experienced progression over a six-year follow-up period, on average. MMD progression can present as worsening stenosis or occlusion in the anterior and posterior cerebral circulation that often results in recurrent brain ischemic events [2].

To restore cerebral blood flow and prevent further ischemic events, surgical revascularization remains the primary treatment for MMD. Encephaloduroarteriosynangiosis (EDAS), considered an indirect revascularization approach, is often employed. But due to the slower development of collateral circulation in adult patients, the effectiveness of EDAS in adults has been questioned [2]. On the contrary, direct revascularization procedures, including superficial temporal artery to middle cerebral artery (STA-MCA) bypass, are able to provide immediate blood flow restoration and show favorable outcomes among adult MMD patients [2].

This report presents the case of a 39-year-old Hispanic female patient with MMD who has experienced multiple ischemic events after EDAS. Her clinical condition necessitated a right craniotomy for STA-MCA bypass. Our case highlights the challenges when managing adults with MMD, as well as the potential need for combined surgical approaches for achieving optimal outcomes.

## Case presentation

Initial evaluation and diagnosis (July 2022)

A 39-year-old female patient with no significant past medical history presented to the Emergency Department (ED) with a four-day history of right-sided numbness and weakness, accompanied by paresthesia in the tongue, right face, arm, and leg.

**Figure 1:**
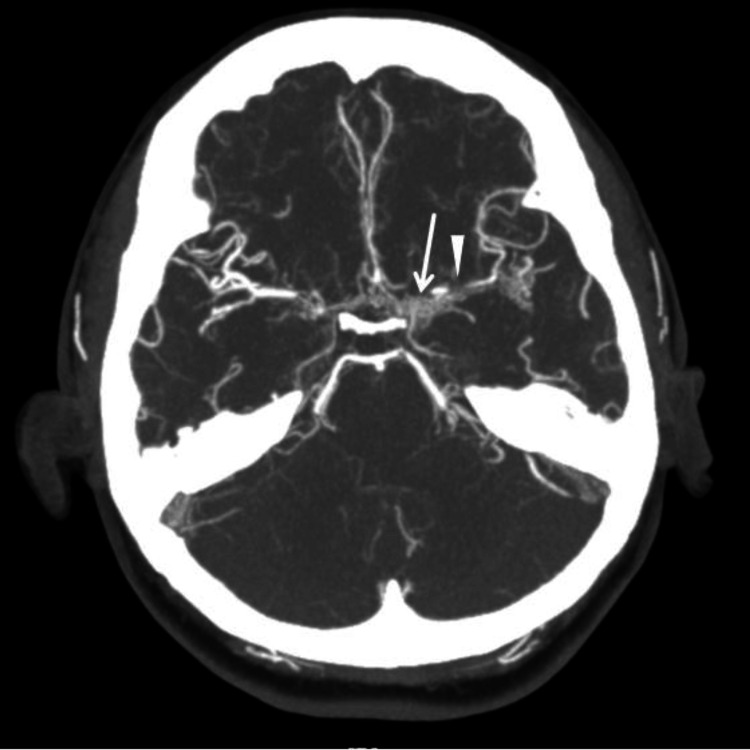
CT angiography of the brain shows a small patent left ICA which becomes subtotally occluded at the carotid siphon (arrowhead) with multiple collaterals around the proximal MCA vessels (arrow). The findings are suggestive of a moyamoya pattern or less likely an unusual vasculitis. ICA: internal carotid artery; MCA: middle cerebral artery

After the initial workups and investigations, brain CT angiography (CTA) suggested a moyamoya pattern or, less likely, an unusual vasculitis (Figure 1). The patient was diagnosed with MMD by a follow-up cerebral angiogram, which confirmed severe advanced moyamoya of the bilateral internal carotid artery termini, extending into the left middle cerebral artery (MCA) M1 segment.

Neurosurgery and neurology teams were consulted, while an immediate surgical intervention was deferred. Patient was initiated on dual antiplatelet therapy with aspirin 81 mg daily and clopidogrel 75 mg daily. Shortly after, she showed clinical improvement, including full resolution of neurological symptoms and return of ambulatory function. She was then discharged home from this hospitalization.

Recurrent episode (November 2022)

In November 2022, the patient had another episode of ischemic event, which featured right arm paresthesia, left facial pain and paralysis, headache, slurred speech, and bilateral visual blurring. Brain CT, MRI, and MRA have been performed, but no new findings were noted. A planned EDAS was scheduled but got postponed due to the patient's newly uncontrolled diabetes. She was treated with a basal-bolus insulin regimen that includes Levemir and Novolog.

EDAS surgical intervention (December 2022)

In December 2022, the patient experienced an episode of difficulty in speaking and perioral paresthesia. This time, she was cleared for patent EDAS of the left STA-MCA, which was performed. However, the patient’s hospitalization was complicated by a left hemispheric infarct postoperatively. The patient's clinical status improved after treatments, and she was discharged home with acetylsalicylic acid (ASA) 81, Plavix 75 mg, and Lipitor 80 mg.

Another ischemic event after EDAS (February 2025)

**Figure 2:**
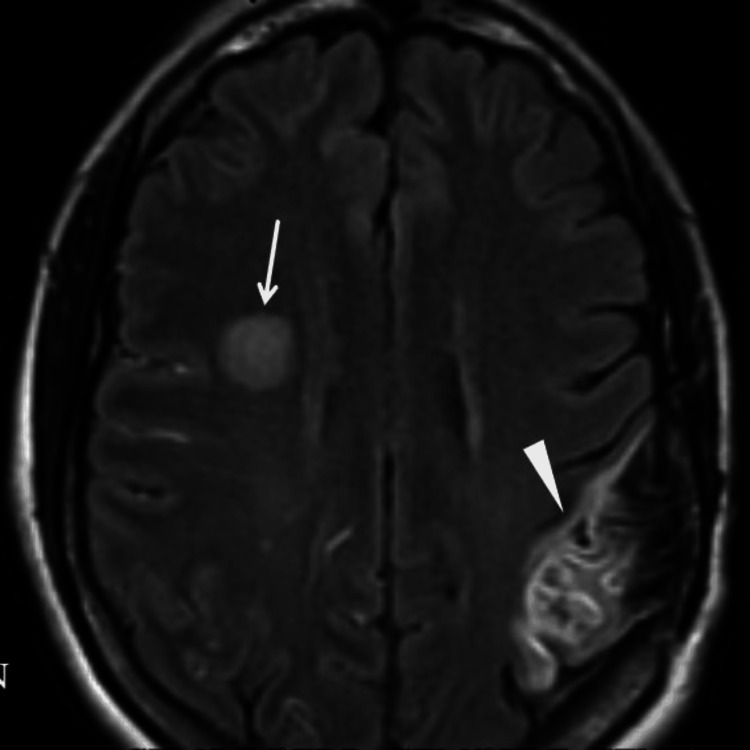
Brain MRI shows a significant lesion in the right centrum semiovale, likely related to an embolic acute ischemic infarct (arrow) and old wedge-shaped hyperintensities in the left parieto-occipital region, suggestive of an old area of ischemic infarct (arrowhead).

About two years later, she returned to the ED presenting with right-sided hand and foot numbness associated with slurred speech for two days. Brain MRI shows a most significant lesion seen in the right centrum semiovale, likely related to an embolic acute ischemic infarct (Figure 2). Neurology onboard, the patient was treated with aspirin 81 mg daily, Lipitor 80 mg daily, and Brilinta 180 mg once, followed by Brilinta 90 mg twice daily. The patient improved clinically and was alert and oriented x 3. Extraocular movements were intact. Speech was clear, with no slurring noted. Cranial nerves were intact. Strength was 5/5 throughout the upper and lower extremities, except 4/5 in the right lower extremity. Sensation was intact throughout. She was cleared by Physical Therapy, Occupational Therapy, the Swallow Team, and Speech Therapy for discharge home independently. The patient was discharged with aspirin 80 mg, Lipitor 80 mg daily, and Plavix 75 mg daily (As Brilinta is not covered by charity care, neurology agreed to discharge with Plavix instead).

Fifth presentation (April 2025)

**Figure 3:**
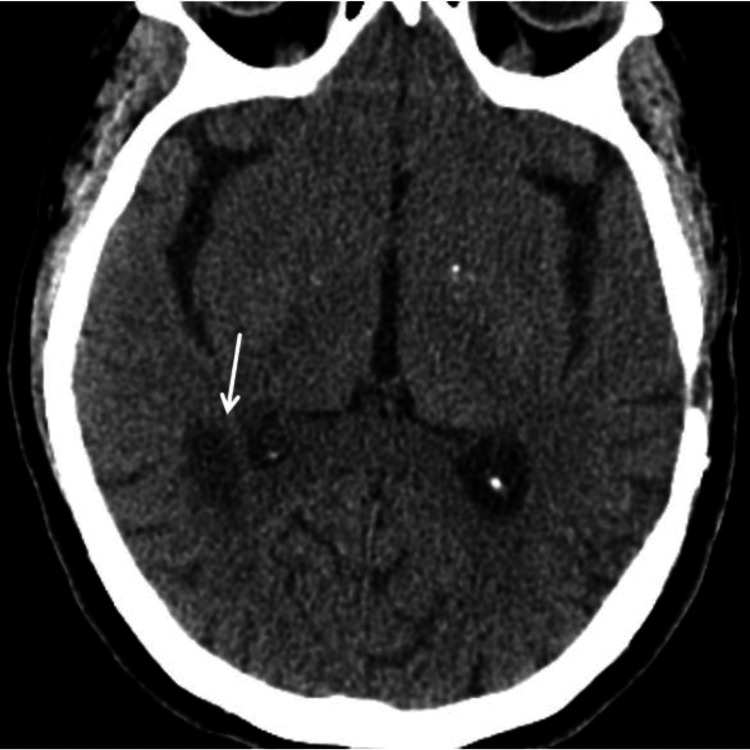
CT brain shows acute to subacute infarct in the right corona radiata and right posterior periventricular region (arrow).

In less than two months, the patient presented again to the ED with new left-sided extremity weakness and expressive aphasia in early April, 2025. Non-contrast brain CT identified an acute-to-subacute infarct in the right corona radiata and right posterior periventricular region (Figure 3). The patient was not eligible for thrombolysis secondary to a recent brain infarct within the past three months, and symptom resolution. Also, CTA revealed a significant 90% stenosis of the right internal carotid artery (ICA) and 80% stenosis of the left ICA. She received the 300 mg loading dose of Plavix and continued with aspirin 81 mg and Plavix 75 mg.

The Neurosurgery team was consulted and onboard, who performed another cerebral angiogram, which showed a new total occlusion of the left posterior cerebral artery from its origin, indicating involvement of the advancing posterior circulation. The progression of the disease in the right posterior cerebral artery P1 segment showed reduced pial collateral flow to the right MCA territory (right parietal and temporal lobes). There was severe right ICA moyamoya with ICA occlusion with runoff to the ophthalmic artery and only moyamoya collaterals. Delayed perfusion of the right MCA (M1-M2) was noted, showing a very late venous phase of the right ICA injections supplied via the moyamoya collaterals. Left ICA advanced moyamoya of the terminus with extensive lenticulostriate collaterals was noted, which indicated the progression to a later Suzuki stage.

The above findings confirmed the patient's significant MMD progression and the need for surgical revascularization. Our Neurosurgery team helped arrange a transfer of the patient to another hospital, where she underwent right STA-MCA bypass via craniotomy.

Ongoing management and follow-up

Following discharge, the patient has been closely followed up with the primary care and neurology teams on long-term aspirin and atorvastatin.

## Discussion

MMD is a rare cerebrovascular disorder characterized by progressive stenosis or occlusion of the terminal ICA, which can lead to the formation of collateral vessels (moyamoya vessels) in order to maintain cerebral perfusion. Even though MMD is more commonly diagnosed among children, adult-onset MMD is a distinct clinical entity, which usually presents with ischemic symptoms with a greater variability in the progression of the disease and the responses to treatments [1,2]. Despite the availability of medical and surgical interventions, MMD can still be challenging to manage, particularly in adult patients.

Our case highlights the unique progression of the MMD in a relatively young Hispanic female patient who continued to experience recurrent ischemic events despite appropriate medical therapies and indirect surgical revascularization with EDAS. The patient’s eventual need for direct revascularization with a right craniotomy for STA-MCA bypass highlights the limitation of indirect revascularization alone and the necessity for a more aggressive surgical approach in some progressive or advanced MMD cases. This clinical course aligns with research suggesting that adult patients with MMD may not achieve long-term stability with EDAS alone [3].

Even though EDAS is a well-established revascularization technique, particularly in pediatric patients, its efficacy in adult patients with advanced MMD remains less clear. The main mechanism by which EDAS improves cerebral perfusion primarily relies on angiogenesis and the gradual development of collateral circulation. However, collateral formation in adults is typically slower and even less reliable, especially among patients with extensive vascular involvement or advanced Suzuki stages [2,4]. So the outcomes of EDAS can be suboptimal for those with extensive vascular involvement or delayed diagnosis. In this patient’s case, the initial surgery did lead to some clinical improvement, but its benefits were not sustained long-term. The patient had continued ischemic events with radiographic evidence of progressive stenosis in both anterior and posterior circulations, which implies that the indirect revascularization alone failed to provide sufficient long-term cerebral blood flow for the patient.

On the other hand, direct revascularization procedures, such as STA-MCA bypass, offer immediate augmentation of cerebral perfusion associated with lower recurrent ischemic event rates among adult MMD patients [5]. There are multiple studies, including meta-analyses, that demonstrate that a direct or combined revascularization approach provides superior stroke prevention compared to the indirect approaches alone in symptomatic adult patients [6,7]. The neurological stabilization following the STA-MCA bypass in this case supports the role of direct revascularization as an effective intervention if the indirect interventions are not sufficient.

The progression of posterior circulation involvement further underscores the severity of this patient’s disease course. Stenosis of the PCA has increasingly been recognized as a marker of advanced MMD and is associated with a higher risk of recurrent ischemic events and poorer clinical outcomes [8]. In this case, the reduction in collateral flow and subsequent PCA occlusion likely contributed to persistent cerebral hypoperfusion and ongoing ischemic vulnerability. These findings highlight the critical importance of close longitudinal imaging surveillance to detect disease progression and guide timely escalation of therapeutic strategies.

This case emphasizes the heterogeneous and unpredictable nature of MMD among adult patients. Even if EDAS remains a valuable revascularization intervention, it is likely not a sufficiently solitary management for adults with advanced or aggressive MMD. Careful monitoring for the disease’s progression, early recognition of treatment failures, as well as timely escalation to direct or combined revascularization interventions, are crucial for optimizing cerebral perfusion and preventing further ischemic injuries among this population of patients.

## Conclusions

Our case report highlights the progressive and complex nature of MMD, alongside the challenges in delivering effective treatment for adult-onset patients, especially those with severe or advanced stages of the disease. This patient continued having recurrent ischemic events even though she received the EDAS procedure and required the right STA-MCA bypass to restore her cerebral blood flow and decrease further stroke risk. So this MMD case adds to the growing evidence that EDAS is beneficial but may not be sufficient for severe or advanced MMD in adult patients. In such cases, consideration of more definitive revascularization approaches may be necessary to achieve sustained cerebral perfusion and reduce the risk of recurrent ischemic events.

We believe that early and individualized treatment strategies are crucial for optimal long-standing clinical outcomes in MMD. The findings of this case also emphasize the need for continuous monitoring and reassessment of the surgical approaches. Moreover, ongoing research on optimal treatment and management strategies for adult MMD patients remains critical to improving long-term outcomes.
